# Summary of marine monitoring results and the prices of fishery products from Fukushima, 1 year after the initial release of “treated water”

**DOI:** 10.3389/fpubh.2024.1527347

**Published:** 2024-12-24

**Authors:** Isamu Amir, Naomi Ito, Masaharu Tsubokura

**Affiliations:** Department of Radiation Health Management, Fukushima Medical University School of Medicine, Fukushima, Japan

**Keywords:** treated water, tritium, health effects of radiation, environmental effects, Fukushima nuclear accident, fishery products prices, unfounded rumor

## 1 Introduction

On August 24, 2024, 1 year had passed since Tokyo Electric Power Company started releasing water containing tritium, known as “treated water,” processed by the Advanced Liquid Processing System (ALPS), from Fukushima Dai-ichi Nuclear Power Station (FDNPS) into the Pacific Ocean. Along with the discharge, some are concerned about the direct health effects of radiation from the “treated water,” while others worry about potential indirect effects on Fukushima's fishery products. Indeed, unfounded rumors about fishery products emerged, anticipating price drops even before the release.

This “treated water,” which contains tritium (^3^H), is kept in tanks within the FDNPS site. This water was released into the Pacific Ocean beginning August 24, 2023. As of November 5, 2024, about 78.3 kilotons of “treated water,” containing ~14.8 TBq of tritium, have been discharged (1). After the initial discharge of “treated water,” few articles have discussed its health effects (2, 3). Moreover, only one article have summarized marine monitoring results and no articles have analyzed the changes in fishery product prices (4). Therefore, we need to review the actual marine monitoring results and actual change in the prices of fishery products from Fukushima. The study aimed to present facts to prevent the spread of further unfounded rumors.

## 2 Finding results and discussion

**Figure 1:**
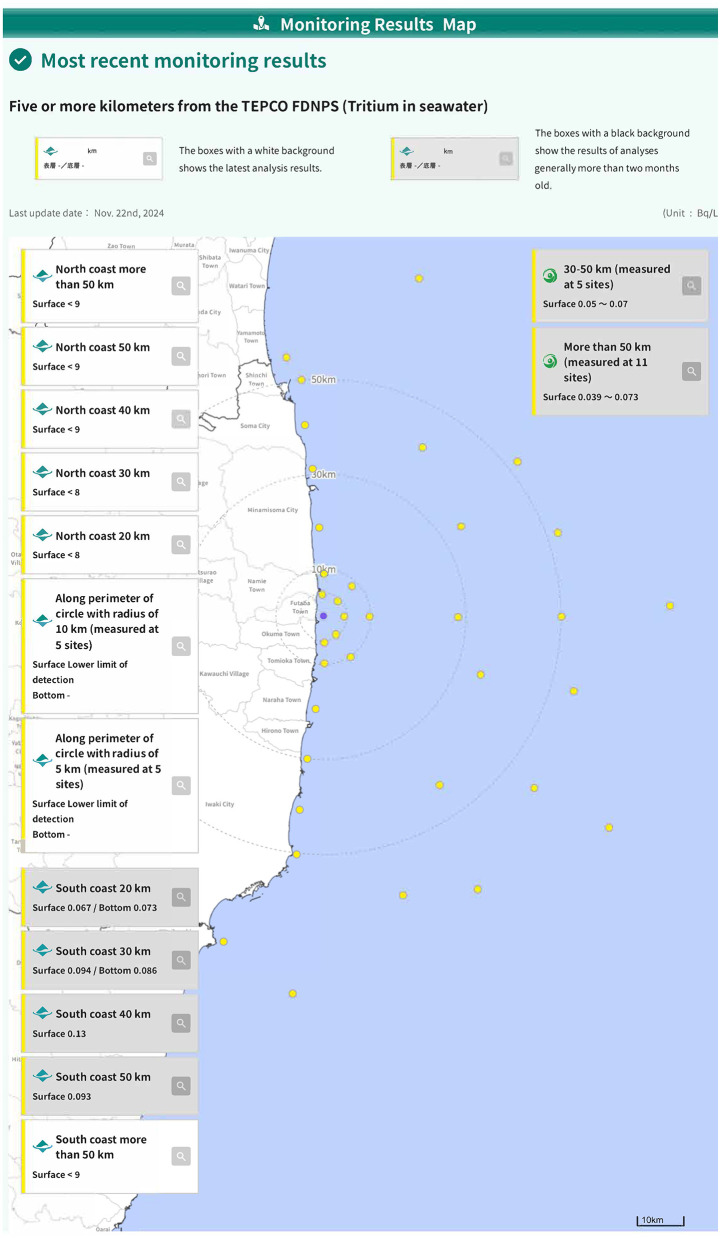
Monitoring results with the discharge of “treated water” from FDNPS, Ministry of the Environment (https://shorisui-monitoring.env.go.jp/en/map/01/).

For marine monitoring, the observation data from the Japanese government have been made public and updated frequently (Figure 1) (5, 6). Tritium concentrations in seawater and other radioactive materials have been continuously monitored within a radius of 400 m to >50 km, from shallow (1.5 m) to deep (>40 m) water, from the FDNPS “treated water” releasing point (Figure 1). Tritium concentrations in live fish, fishery products including algae, and seaweed, and other marine organisms were also monitored before the primary release of “treated water.” The results revealed no adverse effects for humans or the environment were likely to occur because the observed data were either within the range of past fluctuations in tritium concentrations in seawater throughout Japan or below the detection limit (7). Additionally, no changes in tritium concentration levels were observed in live fish and fishery products before and after the release of “treated water” (7).

For assessing actual change in the prices of fishery products, we accessed and collected publicized data of the total amount of fishery products landed throughout Japan and Fukushima, handled in the Tokyo Central Wholesale Market, which deals with the largest amount of fishery products in Japan, from September 2019 to August 2024 (8). We focused on the national average price of fishery products (Yen/kg) and the Fukushima average price, covering 24 months from September 2022, a year before the initial release of “treated water,” to August 2024, using the latest data from their website.

**Figure 2:**
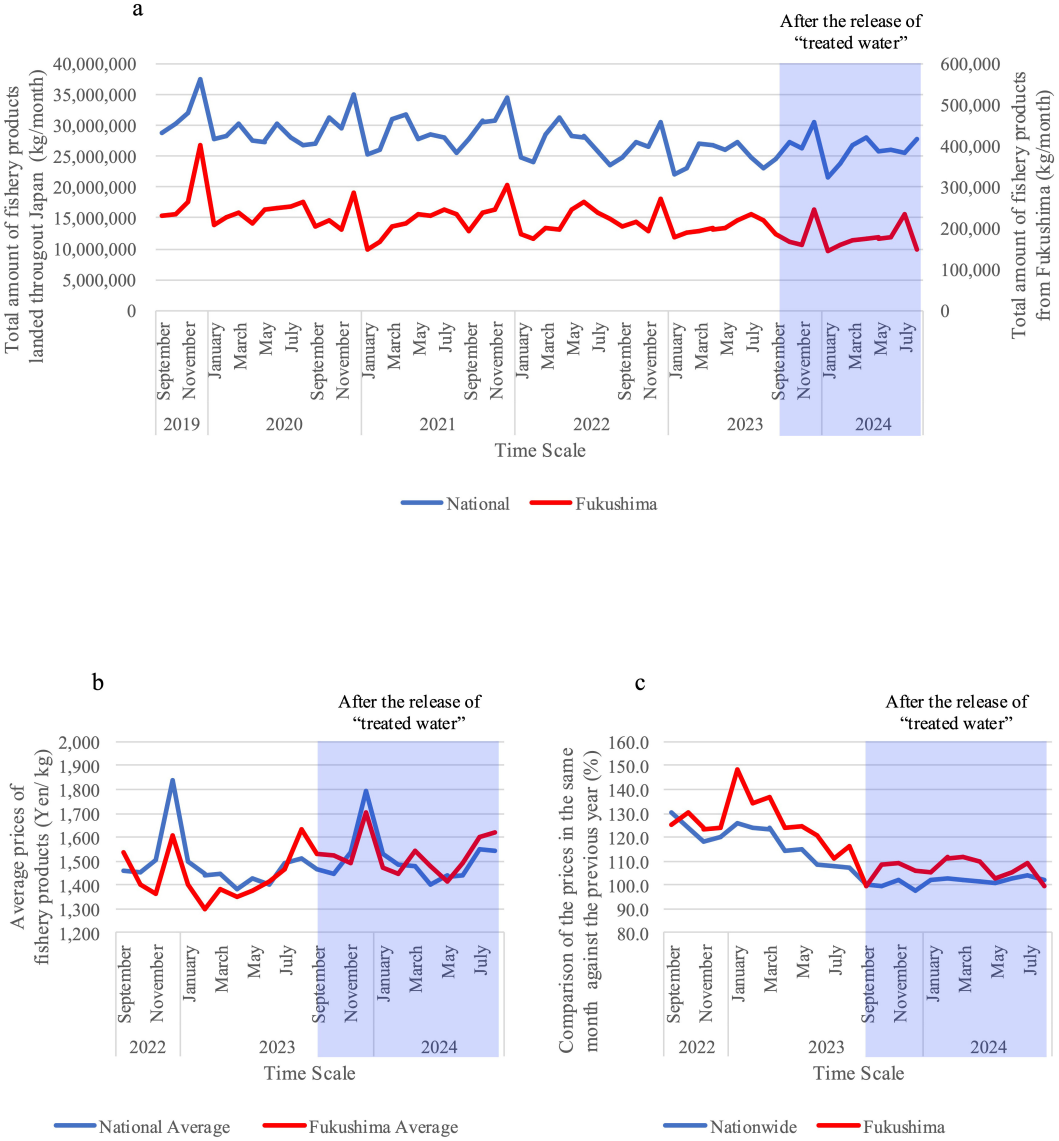
**(A)** Total amount of fishery products, handled in the Tokyo Central Wholesale Market, between those landed throughout Japan (blue) and those from Fukushima (red) from September 2019 to August 2024; **(B)** average prices of fishery products between the national average (blue) and the Fukushima average (red) from September 2022 to August 2024; and **(C)** comparison of the prices in the same month against the previous year of the nationwide average (blue) and the Fukushima average (red) from September 2022 to August 2024.

Figure 2 presents three types of comparisons. Figure 2A compares the total amount of fishery products handled in the Tokyo Central Wholesale Market to those landed throughout Japan (blue) and those from Fukushima (red). Figure 2B contrasts the national average prices of fishery products (blue) with the Fukushima averages (red), while Figure 2C compares the average price in the same month against the previous year. The data show that both the total amount and the average prices of fishery products between the whole of Japan and Fukushima remained constant, indicating no significant decline in the amount or price of fishery products from Fukushima. Additionally, no crucial decline was observed in the year-over-year comparison of the designated months. For the statistical analysis, we performed Spearman's correlation coefficient analysis for the data shown in Figures 2A–C as opposed to determining Pearson's correlation coefficient since the data we obtained is non-parametric. The resulting values were 0.68, 0.66, and 0.90, respectively (Figures 2A–C), which indicates that positive correlation between the national value and Fukushima value for the total amount of fishery products and its prices. Additionally, we carried out ANOVA tests to evaluate the data shown in Figures 2B, C. The *p*-value was 0.16 indicating that there are no significant differences among the four groups, which are national average prices and Fukushima average prices of fishery products 1 year before and after the discharge of “treated water.” Thus, there is no indication that prices of Fukushima fishery products decreased after the release of “treated water.”

These findings are crucial because they suggest that the dealing amount and the average price of Fukushima fishery products did not show a severe decline compared with the equivalent national ones. During the period of the comparison, no statistically significant acute decrease in prices was found compared to the previous year statistically. This result illustrates that fish wholesalers, fish shopkeepers or those working at grocery stores or supermarkets, as well as customers, have understood that Fukushima fishery products are safe for consumption and continue to purchase them. Essentially, the data suggests that consumer behaviors were not influenced by unfounded rumors regarding the discharge of “treated water.”

The most important point to ensure in such scenarios is to disseminate accurate information to a broad audience, including those who may not have access to the latest information, in various approaches including education or official announcements by national and international authorities (9–11). The wider the reach of correct information is, the more people can independently eliminate unfounded rumors. Since “treated water” will continue to be released over the next 30 years, continuous action is necessary to ensure fact-based information is widely accessed. Despite the challenges ahead, we must effectively communicate accurate scientific fact and social data, providing reassurance both domestically and internationally, to eliminate unfounded rumor and to support medium- to long-term risk communication regarding the discharge of “treated water.”
